# The Influence of Cochlear Implant-Based Electric Stimulation on the Electrophysiological Characteristics of Cultured Spiral Ganglion Neurons

**DOI:** 10.1155/2020/3108490

**Published:** 2020-09-06

**Authors:** Na Shen, Lei Zhou, Bin Lai, Shufeng Li

**Affiliations:** ^1^Department of Otolaryngology, Zhongshan Hospital, Fudan University, Shanghai, China; ^2^ENT Institute and Department of Otolaryngology, Eye & ENT Hospital, Fudan University, Shanghai, China; ^3^State Key Laboratory of Medical Neurobiology, Institutes of Brain Science, Fudan University, Shanghai 200032, China; ^4^NHC Key Laboratory of Hearing Medicine (Fudan University), Shanghai, China

## Abstract

**Background:**

Cochlear implant-based electrical stimulation may be an important reason to induce the residual hearing loss after cochlear implantation. In our previous study, we found that charge-balanced biphasic electrical stimulation inhibited the neurite growth of spiral ganglion neurons (SGNs) and decreased Schwann cell density in vitro. In this study, we want to know whether cochlear implant-based electrical stimulation can induce the change of electrical activity in cultured SGNs.

**Methods:**

Spiral ganglion neuron electrical stimulation in vitro model is established using the devices delivering cochlear implant-based electrical stimulation. After 48 h treatment by 50 *μ*A or 100 *μ*A electrical stimulation, the action potential (AP) and voltage depended calcium current (*I*_Ca_) of SGNs are recorded using whole-cell electrophysiological method.

**Results:**

The results show that the *I*_Ca_ of SGNs is decreased significantly in 50 *μ*A and 100 *μ*A electrical stimulation groups. The reversal potential of *I*_Ca_ is nearly +80 mV in control SGN, but the reversal potential decreases to +50 mV in 50 *μ*A and 100 *μ*A electrical stimulation groups. Interestingly, the AP amplitude, the AP latency, and the AP duration of SGNs have no statistically significant differences in all three groups.

**Conclusion:**

Our study suggests cochlear implant-based electrical stimulation only significantly inhibit the *I*_Ca_ of cultured SGNs but has no effect on the firing of AP, and the relation of *I*_Ca_ inhibition and SGN damage induced by electrical stimulation and its mechanism needs to be further studied.

## 1. Introduction

Acoustic signal is transferred by cochlear hair cells from mechanical vibration, and then the signal is transmitted to the auditory cortex via SGNs. Cochlear hair cells in the cochlea are critical for hearing ability [1, 2], and most of the hearing loss is due to irreversible hair cell loss. Cochlear implants can partially replace the function of cochlear hair cells and is the most efficient clinical treatment currently for hearing loss patients. Hybrid cochlear implants, known as the electroacoustic stimulation developed to be used for the patients with high-frequency sensorineural hearing loss and low-frequency residual hearing, has a better clinical effect than the traditional full insertion cochlear implant [3–7]. The use of a shorter, thinner cochlear implant electrode array makes it possible to reduce implantation trauma in the low-frequency region of the cochlea, since the array is only inserted into the basal to the middle part of the cochlea, leaving the apical cochlea intact. When “soft” surgery techniques are used, low-frequency residual hearing can be preserved [8]. It can afford better speech and musical melody recognition than full insertion cochlear implant [6, 9–11].

However, the key point of such benefits in the hybrid cochlear implant depends on the preservation of residual hearing within the implanted inner ear. Unfortunately, more and more clinical researches showed that residual hearing would appear tardive and progressive loss after the hybrid cochlear implant [12–14]. But the mechanism of it was still not clear. Surgical trauma [15], inflammatory, or immune response can be the reasons leading to hair cell death [16]. The formation of fibrosis or new bone growth after implantation can also theoretically cause hair loss by attenuating the traveling wave [17]. But the hearing loss caused by the trauma can be reduced through the optimum-designed implanted electrodes and the enhancement of operate skills [18, 19]. Therefore, the simple trauma cannot explain the mechanism of the tardive and progressive hearing loss.

Electrical stimulation may be an important reason to the residual hearing loss after implantation. Electrical stimulation can promote the neural stem cell's differentiation into neurons and can promote the maturation of newly generated neuron [20, 21], and also can directly excite the SGNs and their peripheral processes, and the residual hair cells in the low-frequency area [22]. Thus, we suppose that during the electrical-acoustic stimulation, the electrical stimulation may spread to the low-frequency area, then excite the cochlear hair cells and SGNs. This excitation can overlap with the acoustic stimulation and induce the excitatory toxicity. Like the noise-induced deafness, the main mechanisms of the noise-induced deafness are the glutamate excite-toxicity, calcium overload, and oxidative stress [23–25]. Electrical stimulation can both induce voltage-gating calcium channels (VDCCs) opening, and the calcium influx and multiple types of VDCCs are involved in the neurite growth inhibition of SGNs [26] induced by the electrical stimulation. The continuous electrical stimulation may cause excessive Ca^2+^ influx and lead to toxic effect which induces SGN death [27]. This was verified by our previous study [26] that charge-balanced biphasic electrical stimulation inhibited the neurite growth of SGNs and decreased Schwann cell density in vitro. But the effect of cochlear implant-based electrical stimulation on the electrophysiological characteristics, the base of neuronal signal transmission, of spiral ganglion neurons is unclear.

In this study, we use the electrophysiological method to study whether cochlear implant-based electrical stimulation can induce the change of the voltage-dependent calcium currents (*I*_Ca_) and the AP firing characteristics of SGNs.

## 2. Materials and Methods

### 2.1. Spiral Ganglion Cultures

Postnatal day 4 (P4) to P6 rat pups of both sexes were provided and bred by the Laboratory Animal Center of the Eye, Ear, Nose and Throat Hospital (Shanghai, China) under routine conditions according to the institute's ethical and environmental guidelines. Dissociated spiral ganglion cultures were prepared as follows. Ganglia were dissected from the rat pups after being sacrificed by decapitation, dissociated with trypsin and collagenase, plated on polyornithine/laminin-coated 4-well or 8-well culture chambers (Nalge Nunc International, Naperville, IL), and maintained in high glucose Dulbecco's Modified Eagle's Medium (DMEM) (Gibco, Grand Island, US) with N2 supplement (Invitrogen, Carlsbad, CA) with 10% fetal bovine serum and fresh insulin (10 *μ*g/ml, Sigma-Aldrich, St. Louis, MO) in a humidified incubator with 37°C, 5.0% CO2. The cultures were placed in the incubator for twenty-four hours, and then maintained for another 48 hr with electrical stimulation to fix for current-clamp experiments.

### 2.2. Electrical Stimulation

**Figure 1:**
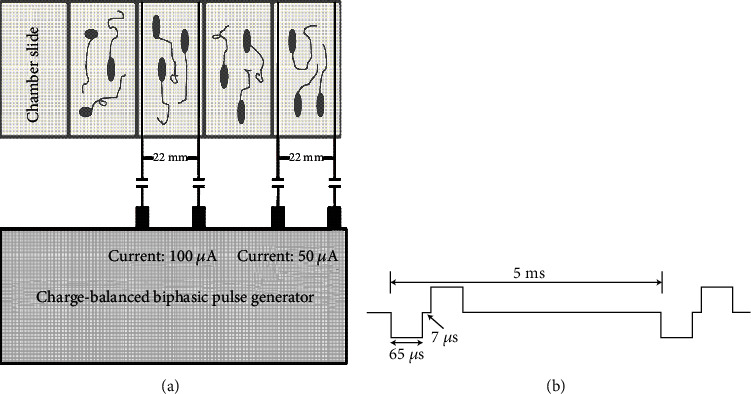
The cultured spiral ganglion neurons (SGNs) are treated with charge-balanced biphasic electrical stimulation. (a) Four-well chamber slides were used in this culture system. Four holes adjacent to the floor were made on two opposite walls of each chamber to introduce two platinum-iridium wires at the two opposite borders. The wires were connected to charge-balanced biphasic pulse generators. (b) The biphasic pluses used for the electrical stimulation were 50 *μ*A or 100 *μ*A amplitude, 65 *μ*s pulse width, 8 *μ*s open-circuit interphase gap, 4862 *μ*s short-circuit phase, and 200 Hz frequency.

Two platinum wires were placed into one well which we wanted to give the electrical stimulation. And then, it should be connected to the artificial cochlea device (Reseat Medical Tech. Co., Ltd, Shanghai, CN) when we gave the electrical stimulation (Figure 1(a)). The parameters for the electrical stimulation were as follows: the pulse width was 65 ms, the frequency was 200 ms (Figure 1(b)), and the current intensity was 50 *μ*A or 100 *μ*A.

### 2.3. Electrophysiology Experiments

Action potential (AP) and voltage-activated Ca^2+^ current (*I_Ca_*) were performed using an Axopatch 200B amplifier (Molecular Devices). Electrodes (4-5 M*Ω*) were pulled from borosilicate glass with P-97 (Sutter, USA). Extracellular solution for APs contained the following (in mM): 145 NaCl, 6 KCl, 2 CaCl_2_, 1 MgCl_2_, 10 Glucose, and 1 HEEPS, at pH 7.3. The tips of the pipettes were filled with the internal solution containing the following (in mM): 133 K-gluconate, 8 NaCl, 0.6 EGTA, 2 Mg·ATP, 0.3 Na3·GTP, and 10 HEPES, at pH 7.3. Extracellular solution for *I*_Ca_ contained the following (in mM): 120 Choline chloride, 20 TEACl, 5 4-AP, 0.02 linopirdine, 2 CsCl, 1.8–5 CaCl_2_, 0.5MgCl_2_, 10 HEPES, and 5 D-glucose, at pH 7.4 with NaOH. The tips of the pipettes were filled with the internal solution containing the following (in mM): 70 CsCl, 70 N-methyl-D-glucamine (NMDG), 1 MgCl2, 10 HEPES, 2–5 EGTA, 1 CaCl_2_, and 4Cs2ATP, at pH 7.2 with CsOH. Recordings were made from neuronal somata at room temperature. Because the cultures contain neuronal and nonneuronal cells, neurons were confirmed by the presence of a large, transient inward sodium current in whole-cell voltage-clamp mode. A holding potential of -70 mV was chosen to assess responses at a level in which there is minimal voltage-dependent ion channel activation. For AP recording, the APs were induced by injecting the current (100 pA, 2000 ms duration) under current clamp mode. *I*_Ca_ current traces were generated with depolarizing voltage steps from a holding potential of -70 mV to 80 mV and stepped to varying positive potentials (*∆* = −10 mV). Whole-cell Ca^2+^current amplitudes at varying test potentials were measured at the peak and steady-state levels using a peak and steady-state detection routine; the current magnitude was divided by the cell capacitance (pF) to generate the current density–voltage relationship. Voltage traces and currents were amplified, filtered (bandpass 2–10 kHz), and digitized at 5–500 kHz using an analog-to-digital convertor Digidata 1200 (Molecular Devices); Data were analyzed using clamp-fit 10.0 software. Data are presented as Mean ± SEM.

### 2.4. Statistical Analysis

Graphs were prepared and statistical analysis was done using GraphPad Prism 5.01 (GraphPad Software, Inc.; CA, USA). The significance of differences among all conditions was compared by unpaired *t*-test and One-way ANOVA Kruskal-Wallis test (*p* < 0.05).

## 3. Results

### 3.1. The Electrical Stimulation Inhibits the Voltage-Dependent Calcium Currents (*I*_Ca_) of SGNs

**Figure 2:**
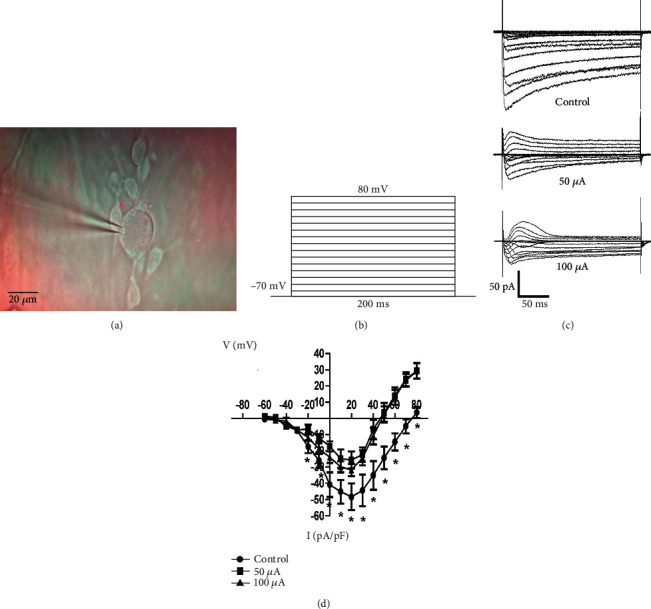
Electrical stimulation decreases voltage-dependent calcium currents (*I*_Ca_) of SGNs. (a) One SGN was patched with the tip of the microelectrode. (b) The stimulation parameters of voltage-dependent calcium current. (c, d) The inhibitory effect of electrical stimulation was obvious when the membrane potential was depolarized with the step ranging between 0 mV and +80 mV. The *I*_Ca_ was activated at -60 mV and reached the peak at 20 mV, the reversal potential of *I*_Ca_ was nearly +75.8 mV ± 4.325 in control neuron, but electrical stimulation could change the reversal potential to +47.5 mV ± 3.497 and +46.3 mV ± 4.369 in 50 *μ*A and 100 *μ*A groups (*p* < 0.05).

The *I*_Ca_ was recorded on the cultured SGNs after different electrical stimulation (Figures 2(a) and 2(b)), and the current-voltage curves of *I*_Ca_ were analyzed. The result showed that the inhibited effect of electrical stimulation on the *I*_Ca_ was obvious when the membrane potential was depolarized from the range between 0 mV and +80 mV. The *I*_Ca_ was activated at -60 mV and reached a peak at 20 mV. From -20 mV to +80 mV, the *I*_Ca_ was significantly decreased in 50 *μ*A and 100 *μ*A groups compared with the control group. The *I*_Ca_ has no difference between 50 *μ*A and 100 *μ*A groups. The reversal potential of *I*_Ca_ was nearly +75.8 ± 4.325 mV in the control group, but after electrical stimulation, the reversal potentials were +47.5 ± 3.497 mV and +46.3 ± 4.369 mV in 50 *μ*A and 100 *μ*A groups correspondingly (Figures 2(c) and 2(d)).

### 3.2. The Influence of Electrical Stimulation on the AP Firing Characteristics of SGNs

**Figure 3:**
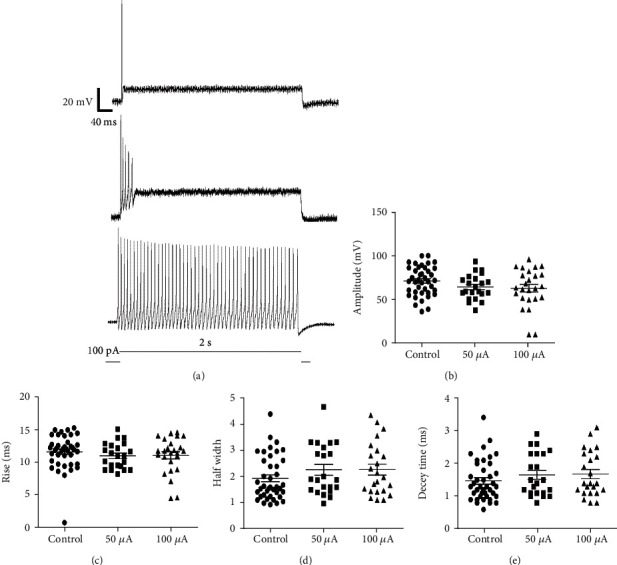
Influence of electrical stimulation on the firing characteristics of SGNs. (a) Type I neuron had only one action potential after electrical stimulation, type II had 2~6 action potentials, and type III could continue firing APs during the test pulse. (b–e) AP amplitude, latency, and duration had no statistically significant differences in all three groups (*p* > 0.05).

A total of 34 SGNs were recorded (control group: *n* = 10; 50 *μ*A group: *n* = 12 and 100 *μ*A group: *n* = 12). Three type action potentials release modes could be found under a depolarized current (100 pA; 2000 ms during) in all neurons; Type I neuron had only one AP after depolarized current stimulation, type II had 2~6 AP and type III could continue firing during the test pulse (Figure 3(a)). The firing characteristics of the first AP were analyzed including AP amplitude, AP decay time, AP half width, and AP rise time. The AP amplitude had no statistically significant difference in all three groups (control group: 69.52 ± 5.880 mV; 50 *μ*A group: 80.48 ± 3.629 mV and 100 *μ*A group: 64.82 ± 3.156 mV). The AP latency (control group: 7.150 ± 0.937 ms; 50 *μ*A group: 8.967 ± 1.292 ms and 100 *μ*A group: 8.054 ± 1.716 ms) and AP duration (control group: 4.142 ± 0.912 ms; 50 *μ*A group: 3.967 ± 0.683 ms and 100 *μ*A group: 3.647 ± 0.677 ms) also had no statistically significant difference in all three groups (Figures 3(b)–3(e)).

## 4. Discussion

In mammal's inner ear, cochlear hair cells and SGNs are two key cell types for hearing ability, Cochlear hair cells convert the mechanical sound vibrations into electronic neural signals, and SGNs transmit these electronic signals to the auditory cortex. In mammal's inner ear, cochlear hair cells and SGNs are sensitive for multiple stress and injuries, including noise, gene mutation, ototoxic drugs, inflammation, and aging [28–31]. On the other hand, the mammal's cochlea only has very limited hair cell and SGN regeneration ability, most of the hair cell loss and SGN loss are permanent and cannot be reversed [32, 33]. Thus, most of the hearing loss is irreversible, and there is no clinical treatment to perfectly cure hearing loss in the clinic by far. Cochlear implant (CI) is an artificial instrument, which is the most widely used neural prosthetic by delivering electrical signals converted from sound information to spiral ganglion neurons and can partially replace the function of cochlear hair cells, and thus is the most efficient clinical treatment currently for hearing loss patients. In the last decade, electric-acoustic stimulation (EAS) technology was newly developed for patients with severe or profound high-frequency hearing loss and residual low-frequency hearing. EAS technology significantly improves music appreciation and speech recognition in background noise through the preservation of residual low-frequency [3, 34]. Unfortunately, clinical trials showed that 30–75% of EAS recipients experienced delayed progressive loss of residual low-frequency hearing over time after the activation of EAS [12–14]. However, the mechanism of this delayed hearing impairment is still not clear by far. In this study, we explored the influence of cochlear implant-based electric stimulation on the electrophysiological characteristics of cultured spiral ganglion neurons.

Calcium overload and oxidative stress are the main causes for SGNs death [27] and the important mechanisms of delayed neuronal death [35]. Our previous study found that charge-balanced biphasic electrical stimulation inhibited the neurite growth of SGNs and decreased Schwann cell density in vitro, and calcium influx through multiple types of VDCCs was involved in the electrical stimulation-induced neurites growth inhibition in SGNs [26]. In this study, we found that *I*_Ca_ was significantly reduced after 48 h electrical stimulation in both 50 *μ*A and 100 *μ*A group. There may be two reasons for the inhibition of *I*_Ca_ caused by electrical stimulation. Firstly, electrical stimulation can decrease voltage-dependent calcium channel expression on the membrane of SGNs, which may be a self-protection mechanism of neurons to reduce the increase of intracellular calcium. Secondly, *I*_Ca_ is directly proportional to the difference of calcium ion concentration inside and outside of the neuron. Electrical stimulation can lead to the release of calcium ions from the intracellular calcium storage; the increase of intracellular calcium concentration can decrease the difference of calcium ion concentration inside and outside of the neuron and leads to the decrease of *I*_Ca_. Although calcium overload and calcium deficiency are the opposite, they can all be caused by the same stimulus, but only in different stage [36, 37]. There are several evidences for calcium deficiency inducing the apoptosis of neuron. Nakamura et al. found that there was no calcium overload of the neurons in the late stage of apoptosis [38] and even the resting calcium level is lower than normal, and the voltage-dependent calcium influx is significantly reduced [39]. Decreasing extracellular calcium concentration or blocking voltage-gated calcium channels can both trigger neuronal apoptosis [40]. Therefore, neuronal survival may depend on appropriate intracellular calcium set points [41]. Excessively high or low calcium is not conducive to the survival of neurons [42, 43]. In conclusion, the change in voltage-dependent calcium current induced by electrical stimulation reflects an imbalance in intracellular calcium homeostasis, which may be a major cause of neuronal death and apoptosis in the later period, although there is no significant abnormality in AP firing in SGNs.

Electrical stimulation generated by CI itself may also be one of the important factors to affect the survival of residual SGNs. Previous studies have reported that the electric conductive biomaterials and the electrical stimulation have very obvious effects on regulating the proliferation and differentiation of neural stem cells [44, 45], as well as on regulating the survival and maturation of neurons, including SGNs [46]. In this study, we found that electrical stimulation could decrease the *I*_Ca_ but had no effect on the action potential firing in cultured SGNs. This result suggests that, although CI can simulate sound stimulation and active the SGNs, but long-term stimulation of CI will break the calcium balance of SGNs and affect the long-term survival of SGNs negatively.

In addition, Schwann cells, as the main glial cells of the peripheral nervous system, have been shown to secrete a variety of nerve growth factors and axon protection factors, such as glial cell line-derived neurotrophic factor (GDNF) and brain Brain-derived neurotrophic factor (BDNF), can promote the growth of nerve cells [47]. Therefore, electrical stimulation may also affect the surrounding Schwann cells, causing them to degenerate and further aggravate the inhibitory effect of electrical stimulation on the growth and function of spiral neurons.

The SGNs are the first-class neurons of the auditory system [48]. Two types of SGNs were found to compose the first neural elements in the auditory pathway [49]. Type I SGNs exhibit input from only one inner hair cell, whereas type II SGNs extend long projections and receive input from dozens of outer hair cell [50]. Reid et al. proved that there were two types of SGNs with different electrophysiological firing pattern. Type I SGNs could be consistently classified as rapidly accommodating at stimulation and firing only one action potential, while type II SGNs fired significantly more action potentials in response to stimulation [49]. In this study, we also found SGNs with one action potential and 2~6 action potential after electrical stimulation. According to the previous reports, these two types of SGNs can be defined. But one type of SGNs that could continue firing was found in this study. Can it be the third type of SGNs? We will confirm this in the further study.

The firing characteristics of the first AP were analyzed including AP amplitude, AP decay time, AP half width, and AP rise time. There were no statistical significance in all the three groups. This may prompt that electrical stimulation can affect the axon length of SGNs [26] by changing the status of calcium ion channel, but it could not change the AP firing of SGNs by affecting the voltage-dependent sodium channel and potassium channel. The axon retraction may damage the intact between SGNs and cochlear hair cells and cause hearing loss.

## 5. Conclusion

Our study suggests cochlear implant-based electrical stimulation only significantly inhibit the *I*_Ca_ of cultured SGNs but has no effect on the release of AP, and the relation of *I*_Ca_ inhibition and SGN damage induced by electrical stimulation and its mechanism needs to be further studied.

## Data Availability

The data used to support the findings of this study are available from the corresponding author upon request.
